# Brain structural correlates of insomnia severity in 1053 individuals with major depressive disorder: results from the ENIGMA MDD Working Group

**DOI:** 10.1038/s41398-020-01109-5

**Published:** 2020-12-08

**Authors:** Jeanne Leerssen, Tessa F. Blanken, Elena Pozzi, Neda Jahanshad, Lyubomir Aftanas, Ole A. Andreassen, Bernhard T. Baune, Ivan Brack, Angela Carballedo, Christopher R. K. Ching, Udo Dannlowski, Katharina Dohm, Verena Enneking, Elena Filimonova, Stella M. Fingas, Thomas Frodl, Beata R. Godlewska, Janik Goltermann, Ian H. Gotlib, Dominik Grotegerd, Oliver Gruber, Mathew A. Harris, Sean N. Hatton, Emma Hawkins, Ian B. Hickie, Natalia Jaworska, Tilo Kircher, Axel Krug, Jim Lagopoulos, Hannah Lemke, Meng Li, Frank P. MacMaster, Andrew M. McIntosh, Quinn McLellan, Susanne Meinert, Benson Mwangi, Igor Nenadić, Evgeny Osipov, Maria J. Portella, Ronny Redlich, Jonathan Repple, Matthew D. Sacchet, Philipp G. Sämann, Egle Simulionyte, Jair C. Soares, Martin Walter, Norio Watanabe, Heather C. Whalley, Dilara Yüksel, Dick J. Veltman, Paul M. Thompson, Lianne Schmaal, Eus J. W. Van Someren

**Affiliations:** 1 Department of Sleep and Cognition, Netherlands Institute for Neuroscience, an Institute of the Royal Netherlands Academy of Arts and Sciences, Amsterdam, The Netherlands; 2 Department of Integrative Neurophysiology, Center for Neurogenomics and Cognitive Research (CNCR), Amsterdam Neuroscience, VU University Amsterdam, Amsterdam, The Netherlands; 3 Melbourne Neuropsychiatry Centre, Department of Psychiatry, The University of Melbourne & Melbourne Health, Melbourne, VIC Australia; 4 Orygen, The National Centre of Excellence in Youth Mental Health, Parkville, VIC Australia; 5 Imaging Genetics Center, Mark and Mary Stevens Neuroimaging and Informatics Institute, Keck School of Medicine of USC, University of Southern California, Marina del Rey, CA USA; 6 Department of Clinical Neuroscience, Behavior & Neurotechnology, Scientific Research Institute of Neuroscience & Medicine, Novosibirsk, Russia; 7 Department of Neuroscience, Novosibirsk State University, Novosibirsk, Russia; 8 NORMENT Centre, Division of Mental Health and Addiction, Oslo University Hospital, Oslo, Norway; 9 Institute of Clinical Medicine, University of Oslo, Oslo, Norway; 10 Department of Psychiatry, Melbourne Medical School, University of Melbourne, Parkville, VIC Australia; 11 Department of Psychiatry, University of Münster, Münster, Germany; 12 Department of Psychiatry, Trinity College Dublin, Dublin, Ireland; 13 Department of Psychiatry and Psychotherapy, Otto von Guericke University Magdeburg, Magdeburg, Germany; 14 Psychopharmacology Research Unit, Department of Psychiatry, University of Oxford, Oxford, UK; 15 Department of Psychology, Stanford University, Stanford, CA USA; 16 Section for Experimental Psychopathology and Neuroimaging, Department of General Psychiatry, Heidelberg University, Heidelberg, Germany; 17 Division of Psychiatry, University of Edinburgh, Edinburgh, UK; 18 Youth Mental Health Team, Brain and Mind Centre, University of Sydney, Sydney, NSW Australia; 19 University of Ottawa’s Institute of Mental Health Research, Ottawa, ON Canada; 20 Cellular & Molecular Medicine, University of Ottawa, Ottawa, ON Canada; 21 Department of Psychiatry and Psychotherapy, Philipps-University Marburg, Marburg, Germany; 22 Department of Psychiatry and Psychotherapy, University of Bonn, Bonn, Germany; 23 Sunshine Coast Mind and Neuroscience Thompson Institute, Birtinya, QLD Australia; 24 Department of Psychiatry and Psychotherapy, Jena University Hospital, Jena, Germany; 25 Max Planck Institute for Biological Cybernetics, Tübingen, Germany; 26 Psychiatry and Paediatrics, University of Calgary, Calgary, AB Canada; 27 Strategic Clinical Network for Addictions and Mental Health, Calgary, AB Canada; 28 Centre for Cognitive Ageing and Cognitive Epidemiology, University of Edinburgh, Edinburgh, UK; 29 Faculty of Medicine and Dentistry, University of Alberta, Edmonton, AB Canada; 30 Department of Psychiatry, University of Texas Health Science Center at Houston, Houston, TX USA; 31 Department of Psychiatry, Institute of Biomedical Research Sant Pau, Barcelona, Spain; 32 CIBERSAM, Barcelona, Spain; 33 Department of Psychology, University of Halle, Halle, Germany; 34 Center for Depression, Anxiety, and Stress Research, McLean Hospital, Harvard Medical School, Belmont, MA USA; 35 Max Planck Institute of Psychiatry, Munich, Germany; 36 UT Center of Excellence on Mood Disorders, Department of Psychiatry and Behavioral Sciences, University of Texas Health Science Center at Houston, Houston, TX USA; 37 Department of Psychiatry and Psychotherapy, University of Tübingen, Tübingen, Germany; 38 Department of Health Promotion and Human Behavior, Graduate School of Public Health/ School of Public Health, Kyoto University, Kyoto, Japan; 39 Center for Health Sciences, SRI International, Menlo Park, CA USA; 40 Department of Psychiatry, Amsterdam UMC, Amsterdam Neuroscience, VU University, Amsterdam, Netherlands; 41 Amsterdam Neuroscience, VU University Medical Center, Amsterdam, Netherlands; 42 Centre for Youth Mental Health, The University of Melbourne, Melbourne, VIC Australia

**Keywords:** Depression, Human behaviour

## Abstract

It has been difficult to find robust brain structural correlates of the overall severity of major depressive disorder (MDD). We hypothesized that specific symptoms may better reveal correlates and investigated this for the severity of insomnia, both a key symptom and a modifiable major risk factor of MDD. Cortical thickness, surface area and subcortical volumes were assessed from T1-weighted brain magnetic resonance imaging (MRI) scans of 1053 MDD patients (age range 13-79 years) from 15 cohorts within the ENIGMA MDD Working Group. Insomnia severity was measured by summing the insomnia items of the Hamilton Depression Rating Scale (HDRS). Symptom specificity was evaluated with correlates of overall depression severity. Disease specificity was evaluated in two independent samples comprising 2108 healthy controls, and in 260 clinical controls with bipolar disorder. Results showed that MDD patients with more severe insomnia had a smaller cortical surface area, mostly driven by the right insula, left inferior frontal gyrus pars triangularis, left frontal pole, right superior parietal cortex, right medial orbitofrontal cortex, and right supramarginal gyrus. Associations were specific for insomnia severity, and were not found for overall depression severity. Associations were also specific to MDD; healthy controls and clinical controls showed differential insomnia severity association profiles. The findings indicate that MDD patients with more severe insomnia show smaller surfaces in several frontoparietal cortical areas. While explained variance remains small, symptom-specific associations could bring us closer to clues on underlying biological phenomena of MDD.

## Introduction

Multiple findings highlight the importance of insomnia for psychiatric disorders in general, and in particular for major depressive disorder (MDD)^1^. Insomnia is a primary risk factor for developing MDD, e.g., ref. ^2^, and its presence in people suffering from MDD hampers the effectiveness of clinical interventions, e.g., ref. ^3^. Treating insomnia can also improve the outcome of patients suffering from depression^4,5^. Moreover, recent genome-wide association studies report a strong genetic correlation between insomnia and depressive symptoms and MDD^6,7^. Given these findings, it seems highly relevant to identify neural correlates of insomnia severity in people suffering from MDD.

To date, brain structural correlates of insomnia symptoms in people with MDD are largely unexplored. Elucidating such correlates may provide key clues to ultimately uncovering the neural correlates of the risk for MDD development. Several anatomical magnetic resonance imaging studies compared people with insomnia disorder (ID) without MDD to those without sleep complaints. People with ID reported smaller gray matter (GM) volumes in the orbitofrontal (OFC)^8–10^, parietal^8^ as well as middle cingulate^11^ cortices, the pineal gland^12^, the thalamus^13^, and a smaller volume and surface area in the inferior frontal gyrus pars triangularis^14^, as well as a larger GM volume in the rostral anterior cingulate cortex (rACC)^9^. Some studies have suggested a smaller hippocampal volume in people with insomnia^15,16^, but these findings could not be replicated, e.g., refs. ^8–10,17^. Other studies in people with ID assessed cortical thickness and found a thinner cortex in the ACC, precentral and lateral prefrontal cortex^18^ and a thicker cortex in several OFC regions, the rACC, middle cingulate cortex, insula, superior parietal cortex, and fusiform area^19^. In MDD patients, brain structural correlates of insomnia severity have hardly been investigated. A larger amygdala and smaller medial OFC have been reported in MDD patients with insomnia^20,21^ as compared to MDD patients without insomnia.

It is tempting to presume that brain areas involved in the severity of insomnia in people without MDD are also involved in the severity of insomnia in people suffering from MDD. However, the complexity of the neuronal networks involved in sleep regulation and MDD makes it also conceivable that different brain mechanisms can underlie seemingly similar sleep complaints^22,23^. The present study therefore applied a whole-brain analysis to uncover brain structural correlates of insomnia severity in people diagnosed with MDD. We evaluated, in a sample of 1053 MDD patients, whether insomnia severity was associated with global and regional differences in cortical thickness, cortical surface areas, and volumes of subcortical regions. Additionally, we evaluated whether the identified associations: (1) were specific to insomnia or driven by overall depression severity and (2) specific to MDD or also present in healthy controls (*n* = 2108) and clinical controls with bipolar disorder (BD; *n* = 260).

## Materials and methods

### Samples

Data for the main analysis were assembled from 15 independent samples of the ENIGMA (Enhancing NeuroImaging Genetics through Mega-Analysis) MDD working group (http://enigma.ini.usc.edu/). We included 1053 people who met criteria for current MDD and had completed the Hamilton Depression Rating Scale (HDRS)^24^. Supplementary Table S1 lists the diagnostic instruments and the exclusion criteria applied at each of the 15 participating sites. Additional data from clinical controls and healthy controls were assembled to evaluate whether insomnia associations were specific to MDD (see Supplementary Methods for details). For clinical controls, we were able to include 260 patients from 5 ENIGMA BD working group sites in whom the HDRS had been assessed (see Supplementary Table S2 for demographics). Next to a first healthy control sample of ENIGMA (*n* = 1277 completed the HDRS), we evaluated associations in a second healthy control sample from the Human Connectome Project (HCP)^25^ of which *n* = 831 had completed the Pittsburgh Sleep Quality Index (PSQI)^26^ (see Supplementary Table S3 for demographics). Exclusion criteria for healthy controls were a history of MDD, a current diagnosis of MDD, or any other psychiatric disorders. All sites obtained approval from local institutional review boards and ethics committees. All participants provided informed consent.

### Severity of insomnia and overall depression severity

Three HDRS items were summed to obtain a valid insomnia severity score^27^ and the remaining items for an insomnia-independent depression severity score, here referred to as the HDRS-14 (Supplementary Methods). In the second healthy control sample (HCP), corresponding PSQI items were summed to obtain an insomnia severity score and an insomnia-independent depression severity score was calculated by excluding the sleep item from the total depression score of the Achenbach Adult Self Report questionnaire^28^. Supplementary Methods provide details and validation.

### Image processing and analysis

Image acquisition parameters for each site are provided in Supplementary Table S4. Schmaal et al. and Glasser et al.^29–31^ provide details of the use of FreeSurfer^32^ segmentation to obtain surface area and thickness of 68 cortical regions^33^, as well as 14 subcortical volumes, lateral ventricle volumes, 2 whole-hemisphere measures, and intracranial volume (ICV).

### Statistical analyses

#### MDD patients

Linear mixed-effects models regressed insomnia severity on surface and thickness of cortical regions and subcortical volume. First, we evaluated whether insomnia severity could be predicted from the overall cortical surface area, its average thickness, or from total subcortical volume. Separate models subsequently evaluated individual brain regions. Models were adjusted for age, sex, scanner site (random intercept), insomnia-independent depression severity, and, for subcortical volumes, total ICV. False discovery rate (FDR)^34^ correction (*p* < 0.05) was applied to correct the *p* values for multiple comparisons for cortical surface areas and thickness, and subcortical volumes (respectively, 68, 68, and 16 comparisons).

Specificity of detected associations for insomnia versus overall depressive symptoms severity was assessed with corresponding models with either overall HDRS-17 depression severity or the HDRS-14 insomnia-independent depression severity as outcome.

Ancillary mixed-effects models including interaction terms (e.g., surface area * age, surface area * sex, surface area * antidepressant use, surface area * depression recurrence, surface area * age of onset of depression) investigated whether the association of insomnia severity with cortical surface area, thickness, or subcortical volume was modified or confounded by age, sex, the use of antidepressant medication, depression recurrence (first versus recurrent episode patients), or age of onset of depression.

To obtain effect size measures for single regressors within multivariable mixed-effects models, we calculated Cohen’s *f*^2^ statistic. Values of 0.02, 0.15, and 0.35, respectively, indicate a small, medium, or large effect.

The proportion of variance in insomnia severity uniquely explained by the significant brain regions (∆*R*^2^) above and beyond the covariates (age, sex, HDRS-14, site, and ICV for subcortical areas) was computed by subtracting the explained variance of a model with only the covariates from the explained variance of the full model (brain area, age, sex, HDRS-14, site, and ICV for subcortical areas) using the MuMln package in R (www.R-project.org).

#### Clinical controls and healthy controls

Within each of the control samples (BD clinical controls and healthy controls), mixed-effects analyses were repeated, including the same covariates and FDR correction. To formally evaluate whether the insomnia-related brain associations found in MDD were similar or different compared to each of the control samples, models including an interaction term were additionally performed, e.g., surface area * disorder (MDD versus BD). Interaction analyses may lack power and require a larger sample size^35^. Therefore, it was additionally evaluated whether adding controls to the ENIGMA MDD sample would alter the effect sizes we found in MDD patients. An increase in effect size would support a similar or even stronger association in controls as found in MDD. A decrease in effect size on the contrary would suggest that controls only add noise or have an opposite association.

## Results

### MDD patients

**Fig. 1:**
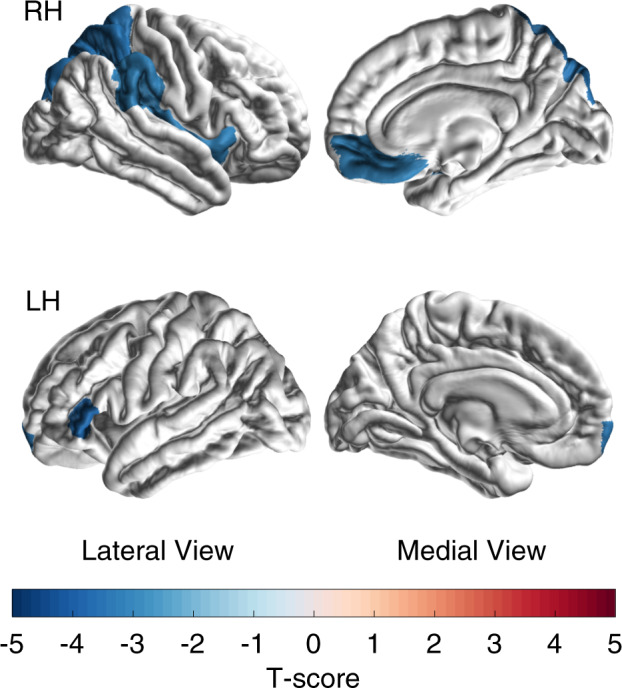
*T*-scores for brain regions that show a significant (*p*_corrected_ < 0.05) decreased surface area associated with higher insomnia severity scores in major depressive disorder patients. Models are adjusted for age, sex, insomnia-independent depression severity (HDRS-14) and site. RH right hemisphere, LF left hemisphere.

Table 1 summarizes the characteristics of the MDD patients included at each site separately and overall. Linear mixed-effects regression indicated more severe insomnia in cases with a smaller total cortical surface area (*f*^2^ = 0.01, ∆*R*^2^ = 0.9%, *p* = 0.044). Table 2, Fig. 1, and Supplementary Fig. S1 provide the results from subsequent mixed-effects regression analyses to investigate which cortical parcels contributed most to this inverse association. In brief, MDD patients with more severe insomnia had smaller surface areas of the right insula (*f*^2^ = 0.02, ∆*R*^2^ = 1.5%, *p*_corrected_ = 0.031), left inferior frontal gyrus pars triangularis (*f*^2^ = 0.02, ∆*R*^2^ = 1.8%, *p*_corrected_ = 0.018), the left frontal pole (*f*^2^ = 0.01, ∆*R*^2^ = 0.6%, *p*_corrected_ = 0.031), right superior parietal cortex (*f*^2^ = 0.02, ∆*R*^2^ = 1.6%, *p*_corrected_ = 0.026), right medial OFC (*f*^2^ = 0.02, ∆*R*^2^ = 1.3%, *p*_corrected_ = 0.031), and the right supramarginal gyrus (*f*^2^ = 0.02, ∆*R*^2^ = 1.3%, *p*_corrected_ = 0.031) (Fig. 1 and Table 2). Together, these brain regions explained 2.7% of the variance in insomnia, above and beyond the variance explained by the covariates. Models including additional covariates (antidepressant medication, depression recurrence, age of onset of depression) did not change the association between surface area and insomnia severity (see Supplementary Results). Ancillary analyses showed that the association between surface area and insomnia severity was not modified or confounded by sex, use of antidepressant medication, depression recurrence, or age of onset of depression. A significant interaction was found between total surface area and age (*p* = 0.046) (see Supplementary Results). The surface area regions we found explain more variance in insomnia severity than they explain variance in overall depression severity (see Supplemental Results and Supplementary Table S5).

**Table 1:** Demographics and clinical characteristics of current MDD patients.

Study	Sample	*N*	Age		% Male	% antidepressant users	% First episode MDD/recurrent MDD	Age of onset MDD		HDRS-17		HDRS-14		HDRS Insomnia	
			Mean	SD				Mean	SD	Mean	SD	Mean	SD	Mean	SD
1	Barcelona	35	47.0	7.1	26	91	51/49	35.1	10.8	19.6	4.5	17.0	4.2	2.6	1.6
2	Calgary	29	17.7	1.8	52	69	0/100	14.1	2.0	20.6	6.8	18.1	6.1	2.6	1.8
3	CLING	27	34.6	11.8	56	100	67/33	31.4	10.6	20.0	4.3	16.7	3.7	3.3	1.4
4	Dublin	52	41.6	10.8	37	71	15/85	25.3	12.8	23.6	5.0	19.6	4.8	4.0	1.5
5	Edinburgh	18	22.9	3.1	33	22	NA	21.4	3.3	5.7^a^	5.8	4.4^a^	4.7	1.3^a^	1.6
6	FOR2017—Marburg	192	37.3	13.5	41	67	17/83	26.5	13.0	9.8	6.3	8.2	5.4	1.6	1.6
7	FOR2017—Münster	29	35.1	13.9	45	83	24/76	25.6	12.8	11.9	7.4	10.0	6.4	1.9	1.6
8	Houston	56	38.6	13.1	27	2	25/71	21.4	11.2	11.7	7.9	9.6	6.5	2.1	2.0
9	Magdeburg	14	39.5	12.0	71	100	21/79	31.6	11.9	12.5	4.0	10.1	4.0	2.4	2.0
10	MPIP	225	46.0	14.0	51	88	34/66	35.7	14.9	24.3	6.2	20.4	5.3	3.9	2.0
11	Münster Neuroimaging Cohort	184	37.3	12.0	45	90	23/77	29.1	12.1	19.0	4.2	15.8	3.6	3.2	1.8
12	Novosibirsk	67	44.7	12.8	27	34	52/48	37.2	14.0	19.0	5.6	16.1	5.1	2.9	1.7
13	Oxford	38	30.1	10.6	37	0	50/50	25.6	9.1	23.0	4.1	18.7	3.6	4.3	1.4
14	Stanford	50	37.2	10.2	42	48	8/88	19.2	8.7	14.6	5.9	12.6	5.2	1.9	1.4
15	Sydney	37	23.2	12.7	19	76	27/73	15.3	6.4	21.6	5.6	18.4	4.5	3.2	1.8
Total		1053	38.6	14.0	41	69	27/71	28.6	13.8	17.9	7.9	15.0	6.8	2.9	2.0

*MDD* major depressive disorder, *HDRS* Hamilton Depression Rating Scale, *NA* not measured.

^a^HDRS scores may be low because they have not been assessed simultaneously with the diagnosis.

**Table 2:** Mixed effect regression analyses estimates of the association of insomnia severity with cortical surface areas (HDRS points/cm^2^) in MDD patients, adjusted for age, sex, insomnia-independent depression severity^a^, and scanning site.

	*B*	s.e.	95% CI	*t*-value	*p* value	FDR *p* value	*N*
Left inferior frontal gyrus pars triangularis	−0.10	0.03	−0.16 to −0.05	−3.66	0.000	0.018	1032
Right superior parietal cortex	−0.03	0.01	−0.05 to −0.01	−3.37	0.001	0.026	1032
Left frontal pole	−0.49	0.15	−0.79 to −0.19	−3.17	0.002	0.031	1051
Right medial orbitofrontal cortex	−0.08	0.03	−0.13 to −0.03	−3.09	0.002	0.031	1032
Right supramarginal gyrus	−0.03	0.01	−0.06 to −0.01	−3.03	0.003	0.031	969
Right insula	−0.06	0.02	−0.10 to −0.02	−3.00	0.003	0.031	1019
Right inferior frontal gyrus pars triangularis	−0.06	0.02	−0.11 to −0.02	−2.73	0.006	0.062	1020
Left insula	−0.06	0.02	−0.10 to −0.01	−2.63	0.009	0.073	1029
Left superior parietal cortex	−0.02	0.01	−0.04 to 0.00	−2.42	0.016	0.118	1027
Right frontal pole	−0.29	0.12	−0.53 to −0.05	−2.36	0.018	0.124	1050
Right paracentral lobule	−0.06	0.03	−0.11 to −0.01	−2.22	0.027	0.167	1046
Left entorhinal cortex	−0.16	0.07	−0.30 to −0.01	−2.14	0.033	0.184	848
Right parahippocampal gyrus	−0.12	0.06	−0.23 to 0.00	−2.05	0.041	0.214	1038
Left parahippocampal gyrus	−0.11	0.06	−0.22 to 0.00	−1.99	0.047	0.227	1036
Right postcentral gyrus	−0.02	0.01	−0.04 to 0.00	−1.95	0.052	0.234	1033
Left posterior cingulate cortex	−0.06	0.03	−0.12 to 0.00	−1.92	0.055	0.234	1046
Right precentral gyrus	−0.02	0.01	−0.04 to 0.00	−1.89	0.059	0.237	1043
Right inferior frontal gyrus pars opercularis	−0.04	0.02	−0.09 to 0.00	−1.84	0.065	0.247	1020
Right superior frontal gyrus	−0.01	0.01	−0.03 to 0.00	−1.75	0.080	0.275	1044
Left precentral gyrus	−0.02	0.01	−0.04 to 0.00	−1.72	0.085	0.275	1033
Right inferior temporal gyrus	−0.02	0.01	−0.04 to 0.00	−1.71	0.087	0.275	1026
Right entorhinal cortex	−0.13	0.08	−0.28 to 0.02	−1.70	0.089	0.275	822
Left inferior frontal gyrus pars opercularis	−0.03	0.02	−0.08 to 0.01	−1.60	0.111	0.328	1034
Right transverse temporal gyrus	−0.14	0.09	−0.32 to 0.04	−1.53	0.127	0.360	1050
Right middle temporal gyrus	−0.02	0.01	−0.04 to 0.01	−1.48	0.139	0.377	1007
Right fusiform gyrus	−0.02	0.01	−0.05 to 0.01	−1.45	0.148	0.384	1030
Left superior frontal gyrus	−0.01	0.01	−0.02 to 0.00	−1.43	0.153	0.384	1034
Left fusiform gyrus	−0.02	0.01	−0.05 to 0.01	−1.41	0.158	0.385	1043
Right precuneus	−0.01	0.01	−0.04 to 0.01	−1.29	0.199	0.440	1046
Left postcentral gyrus	−0.01	0.01	−0.04 to 0.01	−1.25	0.213	0.440	1028
Left supramarginal gyrus	−0.01	0.01	−0.04 to 0.01	−1.23	0.218	0.440	959
Left rostral anterior cingulate cortex	−0.04	0.04	−0.12 to 0.03	−1.23	0.219	0.440	1032
Left inferior frontal gyrus pars orbitalis	−0.08	0.07	−0.22 to 0.05	−1.22	0.224	0.440	1041
Left rostral middle frontal gyrus	−0.01	0.01	−0.02 to 0.01	−1.21	0.225	0.440	1026
Left middle temporal gyrus	−0.02	0.01	−0.05 to 0.01	−1.21	0.228	0.440	978
Right isthmus cingulate cortex	0.04	0.03	−0.03 to 0.11	1.19	0.233	0.440	1049
Left lingual gyrus	−0.02	0.01	−0.04 to 0.01	−1.17	0.241	0.442	1045
Right temporal pole	−0.10	0.09	−0.27 to 0.07	−1.15	0.252	0.443	1029
Left paracentral lobule	−0.03	0.03	−0.09 to 0.02	−1.13	0.257	0.443	1047
Right banks superior temporal sulcus	0.05	0.04	−0.03 to 0.13	1.12	0.263	0.443	990
Right rostral middle frontal gyrus	−0.01	0.01	−0.02 to 0.01	−1.11	0.267	0.443	1030
Right lateral occipital cortex	−0.01	0.01	−0.03 to 0.01	−1.05	0.295	0.478	1037
Right superior temporal gyrus	−0.02	0.01	−0.04 to 0.01	−1.03	0.304	0.480	915
Left medial orbitofrontal cortex	−0.02	0.02	−0.06 to 0.02	−0.97	0.332	0.513	1023
Left isthmus cingulate cortex	−0.03	0.03	−0.09 to 0.03	−0.95	0.342	0.516	1043
Left lateral occipital cortex	−0.01	0.01	−0.03 to 0.01	−0.94	0.350	0.517	1037
Right rostral anterior cingulate cortex	−0.03	0.04	−0.11 to 0.04	−0.91	0.362	0.524	1039
Left inferior temporal gyrus	−0.01	0.01	−0.03 to 0.01	−0.86	0.388	0.549	1009
Right lingual gyrus	−0.01	0.01	−0.04 to 0.02	−0.81	0.420	0.580	1037
Left transverse temporal gyrus	−0.06	0.07	−0.20 to 0.08	−0.80	0.427	0.580	1050
Left inferior parietal cortex	−0.01	0.01	−0.02 to 0.01	−0.74	0.460	0.614	1011
Left pericalcarine cortex	−0.02	0.02	−0.06 to 0.03	−0.71	0.475	0.620	1013
Left precuneus	−0.01	0.01	−0.03 to 0.02	−0.69	0.491	0.620	1042
Left caudal anterior cingulate cortex	−0.03	0.04	−0.10 to 0.05	−0.69	0.492	0.620	1045
Right inferior parietal cortex	0.01	0.01	−0.01 to 0.02	0.64	0.520	0.642	1019
Right caudal anterior cingulate cortex	−0.02	0.03	−0.09 to 0.05	−0.61	0.542	0.650	1042
Left superior temporal gyrus	−0.01	0.01	−0.04 to 0.02	−0.61	0.545	0.650	915
Right caudal middle frontal gyrus	−0.01	0.01	−0.04 to 0.02	−0.59	0.555	0.651	1039
Left cuneus	0.01	0.03	−0.04 to 0.06	0.51	0.607	0.700	1010
Right inferior frontal gyrus pars orbitalis	−0.02	0.06	−0.13 to 0.09	−0.34	0.736	0.834	1038
Left banks superior temporal sulcus	0.01	0.04	−0.06 to 0.08	0.31	0.755	0.842	961
Right pericalcarine cortex	0.01	0.02	−0.04 to 0.05	0.28	0.780	0.856	1010
Left caudal middle frontal gyrus	0.00	0.01	−0.03 to 0.03	−0.23	0.820	0.875	1036
Left lateral orbitofrontal cortex	0.00	0.02	−0.04 to 0.03	−0.22	0.826	0.875	1045
Right lateral orbitofrontal cortex	0.00	0.02	−0.03 to 0.04	0.21	0.836	0.875	1047
Right cuneus	0.00	0.03	−0.06 to 0.05	−0.12	0.907	0.934	1017
Right posterior cingulate cortex	0.00	0.03	−0.06 to 0.06	0.06	0.953	0.967	1048
Left temporal pole	0.00	0.09	−0.17 to 0.17	0.04	0.967	0.967	1025

*MDD* major depressive disorders, *HDRS* Hamilton Depression Rating Scale, *CI* confidence interval, *FDR* false discovery rate.

^a^Insomnia-independent depression severity is calculated by subtracting the insomnia scores from the total HDRS score.

Insomnia severity was not associated with average (*p* = 0.174) or regional cortical thickness (all *p*_corrected_ > 0.574), nor with total (*p* = 0.595) or local subcortical volume (all *p*_corrected_ > 0.886; see Supplementary Tables S6 and S7).

Linear mixed-effects regression models with overall depression severity (HDRS-17) or adjusted depression severity (HDRS-14) as outcome measures revealed no significant predictive value of total (all *p* > 0.300) or regional surface area (all *p*_corrected_ > 0.608), nor for overall average (all *p* > 0.568) or local cortical thickness (all *p*_corrected_ > 0.810), or total (*p* > 0.354) or local subcortical volume (all *p*_corrected_ > 0.238).

### Clinical controls and healthy controls

In BD clinical controls, insomnia severity was not significantly associated with any of the six surface areas found in MDD (all *f*^2^ < 0.01, *p* > 0.205), neither with any of the other local surface area, thickness, or subcortical volume measures (all *p*_corrected_ > 0.984). To formally evaluate whether the association between insomnia severity and surface areas differed between MDD and BD patients, interaction analyses were performed for each of the six surface areas found in MDD and type of disorder (MDD versus BD). A significant interaction effect was found in only 2 out of the 6 surface areas (left inferior frontal gyrus pars triangularis, *p* = 0.022; right supramarginal gyrus, *p* = 0.045), indicating that a smaller surface in these two areas was associated with higher insomnia severity specifically in MDD patients but not in BD patients. When combining the BD and MDD sample (*n* = 1313), the effect sizes decreased by 29–71% as compared to the effects found for cortical surface area in MDD only.

In the ENIGMA-MDD healthy controls, insomnia severity was not significantly associated with any of the six surface areas found in MDD (all *f*^2^ < 0.01, *p* > 0.193), neither with any of the other global or local surface area, thickness, or subcortical volume measures (all *p*_corrected_ > 0.441). When adding the ENIGMA healthy controls to the MDD sample (*n* = 2330), the effect sizes decreased by 34–64% with respect to the significant effects found for cortical surface area in MDD only. In the HCP healthy controls, insomnia severity was only significantly associated with 1 out of the 6 surface areas found in MDD (right medial OFC, *f*^2^ = 0.008, *p* = 0.009; other regions *f*^2^ < 0.002, *p* > 0.188). No significant association was found for any of the other local surface areas or for subcortical volumes (all *p*_corrected_ > 0.089), whereas a significant association was found for 2 out of 68 cortical thickness regions. Healthy controls with more severe insomnia showed a thicker right rACC (*p*_corrected_ = 0.042) and a thinner right entorhinal cortex (*p*_corrected_ = 0.042). Although none of six surface area by group interaction effects reached significance (*p* > 0.074), interaction effects for the two identified cortical thickness regions did (all *p* < 0.016), supporting specificity for these regions to healthy controls but not for MDD patients.

Together these results suggest differential association profiles of cortical measures in MDD that in general do not generalize to BD clinical controls or healthy controls.

## Discussion

This large-scale study investigated brain structural correlates of insomnia severity in MDD and revealed more severe insomnia in cases with a smaller total surface area. This inverse association with total surface area was mostly driven by the right insula, left inferior frontal gyrus pars triangularis, left frontal pole, right superior parietal cortex, right medial OFC, and right supramarginal gyrus, that all showed significant regional effects. The association was independent of depression severity adjusted for the three insomnia items, and was specific for surface area: no associations were found for cortical thickness or subcortical volumes. The association between surface area and insomnia severity seems specific to MDD patients, since no associations were found in healthy or clinical controls. Cortical surface area only explained a small proportion of the variance in insomnia severity, which may not be surprising, because it is conceivable that a variety of other factors influence the complex trait of insomnia. On the other hand, small effects in large samples are more likely to be reliable and reproducible than large effect in small samples^36^.

We found that surface area was specifically associated with insomnia severity, not with overall depression severity. Our meta-analysis^29^ in a large overlapping sample of adult MDD patients and controls from ENIGMA MDD reported no significant association between cortical surface area and depression severity measured using the total score of the HDRS. A weak negative association was only found between self-reported depression severity (Beck’s Depression Inventory, BDI-II score) and surface areas of the bilateral precuneus, left frontal pole, and left postcentral gyrus. Our current findings indicate a better association of total and regional surface areas for the severity of a single-domain phenotype (insomnia symptoms) than for the severity of a multi-domain phenotype (all/other mixed symptoms of depression). It should be noted that the explained variance is still small, as is commonly found across genetic and neuroimaging regressors for complex traits like insomnia and depression. While the findings thus do not explain much of individual differences, they may bring us a bit closer to clues on underlying biological phenomena involved.

The Research Domain Criteria approach to psychiatric disease stresses the importance of identifying fundamental symptom dimensions tied to neural systems that cut across heterogeneous mental disorder classifications^37^. Our findings are the first to identify brain structural correlates related to insomnia, an important clinical symptom of the Arousal and Regulatory Systems domain^38^, in people suffering from MDD. Notably however, these correlates do not seem to cut across disorders.

Our findings indicate that only cortical surface area is predictive of insomnia severity in MDD, whereas cortical thickness and subcortical volume had no predictive value. Prior studies have shown that these measures represent distinct biological processes. For example, cortical surface area, cortical thickness, and GM volume differ in terms of developmental trajectory^39^, network topology^40^, and genetic influences^41^. As compared to cortical thickness, surface area is more strongly determined by genetic influences^42^. To identify common genetic variants that underlie these genetic influences on brain structures is not straightforward, as their effects are very small. To overcome this difficulty, >50 ENIGMA sites recently generated a very large sample (*n* = 35,660) to uncover genetic loci that affect cortical surface area and thickness^42^. The study revealed many loci where variants were associated with surface area. Most interestingly, genetic correlations indicated that the variants associated with a smaller global surface area overlapped more with the variants involved in insomnia^6^ than with variants of any other included symptom or disorder. In light of (1) the strong genetic correlation between insomnia and cortical surface area, (2) the genetic heritability of surface area, and (3) the more externally driven variability of cortical thickness, we consider it likely that overlapping neurobiological mechanisms predispose to both a smaller cortical surface area and more severe insomnia symptoms in MDD. We cannot fully exclude, however, the possibility that insomnia causes a reduction of cortical surface area as secondary process.

We found smaller surface areas of several cortical regions to be associated with insomnia severity in MDD patients; such associations were, however, not found in non-depressed samples. Few studies investigated cortical surface area in relation to insomnia complaints. Lim et al.^43^ found that sleep fragmentation was nominally associated with lower surface area in the banks of the superior temporal sulcus and pars orbitalis. While we did not find cortical thickness to be associated with insomnia severity in MDD, we did find insomnia severity to be associated with thickness alterations in the entorhinal cortex and the rACC in our healthy control sample. Several studies have reported an association between thickness and insomnia severity in non-depressed people^43–45^. More specifically within insomnia patients, one study found thinning in the ACC, precentral cortex, and lateral prefrontal cortex^18^, while in contrast another study found thickening in several areas, including the orbital frontal cortex, rACC, middle cingulate cortex, insula, superior parietal lobule, and fusiform area^19^. Concertedly, these findings provide support for a double dissociation suggesting a depression-specific association of insomnia severity with cortical surface area and an association of insomnia severity with cortical thickness in non-depressed people.

Reduced surface area of the medial OFC, however, was found to be related to insomnia severity in both MDD patients and in healthy controls in our study. One study found reduced GM in the medial OFC in co-morbid depression and insomnia patients compared to insomnia or depressed patients without comorbid disorders^21^. Alterations in the medial OFC might have a symptom-specific role that is similar in in both insomnia and depressed patients.

The cortical regions for which a smaller surface area predicted more severe insomnia are involved in a wide range of functions, including emotional processing (medial OFC, frontal pole, insula), attentional processing and interoceptive awareness (insula), and cognitive control (inferior frontal gyrus pars triangularis, insula, parietal regions)^46,47^. It may—at first glance—be surprising that insomnia severity is significantly associated with the surface area of regions that are primarily involved in these processes, while overall depression severity is not. Recent insights, however, suggest that insomnia involves altered emotion regulation and interoception rather than deficits in sleep regulation per se^48–50^, which is again supported by our findings of reduced surface area in regions involved in emotional processing.

The current study has several limitations. First, we had limited information on sleep in our sample: only three HDRS items about insomnia. It would have been interesting to evaluate whether cortical surface areas showed similar associations with other measures of sleep, as could be derived from sleep diaries, actigraphy, or polysomnography. Even so, actigraphic and polysomnographic measures of sleep hardly correlate with the subjective complaints that diagnostically define insomnia^51^. By contrast, subjective complaints recorded in sleep diaries strongly correlate with the insomnia items of the HDRS^27^. Second, the characteristics of the HCP healthy controls were somewhat different: they were younger, scanned on a different scanner, and asked different insomnia questions than in the ENGIMA MDD sample. Nevertheless, these results still provide valuable insight into how insomnia-related brain alterations may be different in people with MDD than in people without MDD. Third, poor sleep quality might be associated with obstructive sleep apnea, a late chronotype, and sleep duration. Sleep apnea and chronotype have been associated with less GM^52^ and a thinner cortex^53–55^; however, as far as we know no studies have associated these variables with cortical surface area. Insomnia severity might also be associated with sleep duration; however, in a large study of MDD patients the shared variance between insomnia severity and sleep duration was limited (20%)^56^, suggesting discernable dimensions of sleep. Unfortunately, sleep apnea, chronotype, and sleep duration were not systematically assessed in our sample. It would be interesting to evaluate whether our findings are better explained by these variables than by quality of sleep. Lastly, other variables could potentially have contributed to individual differences in our dataset, such as handedness^57^, oral contraceptive use^58^, medical comorbidities, or dementia^59–63^. Future studies could take these variables into account. A major strength of our study is that we obtained data from a large representative sample of MDD patients from 15 different sites, supporting the robustness and generalizability of our results. The robustness of our findings is further supported by the lack of interaction effects of surface area with antidepressant use, depression recurrence, or age of onset of depression.

In conclusion, our study showed that insomnia is more severe in patients with MDD who have a smaller cortical surface area, in particular of the right insula, left inferior frontal gyrus pars triangularis, left frontal pole, right superior parietal cortex, right medial OFC, and right supramarginal gyrus. The better specificity of these associations with insomnia severity than with total depression severity highlights the possibility that insomnia could represent a symptom cluster of MDD with a distinct neurobiological underpinning.

## Supplementary information

supplemental material

## Data Availability

Analysis scripts are available upon reasonable request by contacting the corresponding author.
