# Supplementary material for: Brain structural correlates of insomnia severity in 1053 individuals with major depressive disorder: results from the ENIGMA MDD Working Group
Source: Transl Psychiatry. 2020 Dec 8;10:425. doi: 10.1038/s41398-020-01109-5 (PMC7723989; doi:10.1038/s41398-020-01109-5)
Supplement: Supplementary file 1 — supplemental material [file 41398_2020_1109_MOESM1_ESM.doc]

**Supplemental methods**

**Participants**

**Clinical controls.** Data from 260 individuals with bipolar disorder were included from the ENIGMA Bipolar Disorder working group. Participants were included in the analyses, from whom MRI and HDRS data were available, and who were 13 years or older in order to maintain the same age range as in the ENIGMA MDD sample. We obtained data from 5 cohorts (Edinburgh, FOR2107 Marburg, FOR2107 Münster, Houston, and Münster Neuroimaging Cohort). Table S4 provides the MRI acquisition parameters for each of the sites. Importantly, these 5 samples from the ENIGMA BD working group also contributed data to the ENIGMA MDD samples included in this study and, therefore, were scanned with the same MRI scanners.

**Healthy controls.** A first control sample consisted of the N=1277 healthy volunteers of ENIGMA that had completed the HDRS. We moreover evaluated associations in a second healthy control sample from the Human Connectome Project (HCP) ^1^ of which N=831 had completed the Pittsburgh Sleep Quality Index (PSQI) ^2^ (see Table S3 for demographics). Included HCP participants did not report to having a clinical diagnosis of MDD and/or a major depressive episode in the past according to the Semi-Structured Assessment for the Genetics of Alcoholism (SSAGA) psychiatric interview ^3^. T1 weighted scans were collected on a 3 Tesla scanner (Siemens, Skyra, Erlanger, Germany) with a 32-channel head coil (voxel size = 0.7mm^3^ isotropic, Field of View = 224 mm, matrix = 320, 256 slices, repetition time (TR) = 2400ms, Echo Time (TE) = 2.14ms). Cortical surface area, cortical thickness and subcortical volumes were extracted from 68 cortical and 14 subcortical brain regions according to the protocols described in Glasser et al. (2013) ^4^.

**Severity of insomnia and overall depression severity**

In all ENIGMA samples (MDD, BD clinical controls, and ENIGMA healthy controls), the HDRS questionnaire was used to assess insomnia severity and overall depressive symptoms severity. Insomnia severity was calculated by summing up the scores of item 4, 5 and 6 of the HDRS scale (trouble falling asleep, maintaining sleep, and early morning awakenings). This sum score ranged from 0 to 6, with a higher score indicating more severe insomnia symptoms. The validity of the insomnia items of the HDRS as a measure of insomnia severity is supported by their strong correlations with sleep diary data ^5^. An insomnia-independent depression severity score was calculated by excluding the 3 insomnia severity items from the HDRS total score calculated over 17 items (additional items of the HDRS-21 assessed by some sites were not used). We will refer to the insomnia-independent depression severity score as HDRS-14.

In the HCP healthy control sample, corresponding items on difficulties initiating sleep, maintaining sleep and early morning awakening were assessed from PSQI items 5a and b, summing up to a range from 0 to 6.

We verified that the PSQI-derived insomnia severity rating was highly correlated with the HDRS-derived insomnia severity rating (r = 0.704, p < 0.001) in a pooled sample of 212 participants from three independent studies where both scales had been assessed (study 1: 37 MDD patients with comorbid insomnia from Watanabe et al. (2011) ^6^; study 2: 59 MDD patients, 19 controls, from Enneking et al. (2019) ^7^; study 3: 65 MDD patients, 32 controls, from the ENIGMA-MDD Novosibirsk cohort). An insomnia-independent depression severity score was calculated by excluding one sleep-related item (“I have trouble sleeping”) from the total depressive problems score of the Achenbach Adult Self Report questionnaire ^8^.

**Supplemental results**

**Effects of additional covariates**

Models including additional covariates, antidepressant medication (yes/no), depression recurrence (recurrent or first onset depression), and age of onset of depression, did not change the association between surface area and insomnia severity. The association between insomnia severity and total surface area, right insula, left inferior frontal gyrus pars triangularis, the left frontal pole, right superior parietal cortex, right medial orbitofrontal cortex, remained significant (all p_corrected_ < 0.05). Two additional brain regions showed a significant association between surface area and insomnia severity (left insula, p_corrected_ = 0.040; right inferior frontal gyrus pars triangularis, p_corrected_ = 0.033), after including the following additional covariates: antidepressant medication, depression recurrence, age of onset of depression. The surface area of the right supramarginal gyrus did not show significance with the additional covariates in the model (p_corrected_ = 0.067). Due to missing values on the additional covariates, the above analyses were based on a smaller sample (N = 967-999).

**Interaction effects with age, sex, use of antidepressant medication, depression recurrence or age of onset of depression**

The association between the surface areas of the significant regions (total surface area, the right insula, left inferior frontal gyrus pars triangularis, the left frontal pole, right superior parietal cortex, right medial orbitofrontal cortex, and the right supramarginal gyrus) were not modulated or confounded by sex, use of antidepressant medication, depression recurrence or age of depression onset, all p > 0.218. A significant interaction was found between total surface area and age (p = 0.046), showing a stronger negative association between surface area and insomnia severity in younger people than in older people. The surface areas of the other significant brain regions were not modulated by age, all p > 0.084.

**Comparison of effect sizes for the association with surface area for insomnia severity, and depression severity**

The surface area of all significant regions we found in MDD patients (total surface area, the right insula, left inferior frontal gyrus pars triangularis, the left frontal pole, right superior parietal cortex, right medial orbitofrontal cortex, the right supramarginal gyrus) explain more variance in insomnia severity, than they explain variance in overall depression severity, see Supplementary Table S5. Only 2 out of 7 surface areas (left inferior frontal gyrus pars triangularis, right insula) explain some variance in depression severity (0.1%, 0.3% respectively), but the explained variance of these regions for insomnia severity is substantially higher (1.5%, 1.8% respectively). The size of the effect, reflected in Cohen’s f2, was also higher for insomnia severity, than the effect sizes for depression severity, see Supplementary Table S5.

**Supplementary tables and figures**

| Supplementary Table S1. Instruments for diagnosing Major Depressive Disorder and exclusion criteria by site | | |
| --- | --- | --- |
| Sample | Instrument for diagnosing MDD | Exclusion criteria |
| Barcelona | CIDI-interview | Axis I comorbidity according to DSM-IV-TR criteria was an exclusion criteria for all participants. |
| Edinburgh | SCID interview | MDD subjects: presence of other axis I diagnoses, any major neurological disorder, learning disability, or any history of head injury that included loss of consciousness and any contraindications to MRI. |
| Calgary | KSADS | Dalhousie Sample: A history of neurological illness, medical illness, claustrophobia, >21 year of age, or the presence of a ferrous implant or pacemaker. University of Calgary: Left handed; history of seizures, epilepsy or other neurological or psychiatric diagnoses (specifically bipolar disorder, psychosis, pervasive developmental disorder, eating disorders, PTSD); pregnancy. |
| CLING | ICD-10 interview | Exclusion criteria for MDD subjects were neurological and severe other medical conditions (in particular those that could be related to affective symptoms), lifetime diagnosis of substance dependence, substance abuse during the last month, cannabis abuse during the last 2 weeks, mental retardation as well as past or actual presence of other axis I diagnoses with exception of anxiety disorders. |
| Dublin | SCID-1 interview | MDD subjects: comorbid psychiatric disorders (Axis I or Axis II, other than MDD), Treatment with antipsychotics or mood stabilizers, age 65; history of neurological or other severe medical illness, head injury or severe substance abuse in their lifetime history and general MRI contraindications. |
| FOR2107 | SCID-1 interview | Inclusion criteria: age 18-65 years; patients were diagnosed with major depressive disorder by SCID-Interview, currently depressed or remitted. Exclusion criteria all: any MRI contraindications; any neurological abnormalities; substance dependence or current benzodiazepine treatment (wash out of at least three half-lives before study participation). |
| Houston | SCID interview | MDD subjects: age below 18; lifetime or current diagnosis of psychotic disorder, or bipolar I or II disorder; substance abuse/dependence in 6 months prior to study inclusion; current major medical problems; MRI contra-indications. |
| Magdeburg | ICD-10 interview | MDD subjects: history of seizures, medication with glutamate modulating drugs (ketamine, riluzole, etc.) or benzodiazepines, prior electroconvulsive therapy (ECT) treatments and pregnancy, atypical forms of depression, any additional psychiatric disorder, and a history of substance abuse or dependence; contraindications against MRI, major medical and neurological illness. |
| MPIP | M-CIDI/SCAN interview | 1. Munich Antidepressant Response Signature (MARS) study  MDD subjects (clinical consensus diagnosis or M-CIDI (since 2008)): depressive syndromes secondary to any medical or neurological condition (e. g., intoxication, drug abuse, stroke), the presence of manic, hypomanic or mixed affective symptoms, lifetime diagnosis of alcohol dependence, illicit drug abuse or the presence of severe medical conditions (e.g., ischemic heart disease). Patients with bipolar depression were excluded for the current MR study.  2. Recurrent unipolar depression (RUD) study: MDD subjects (SCAN interview): presence of manic episodes, mood incongruent psychotic symptoms, the presence of a lifetime diagnosis of intravenous drug abuse and depressive symptoms only secondary to alcohol or substance abuse or to medical illness or medication. All subjects: gross incidental MR findings such as territorial infarction, tumor, hydrocephalus, malformations and anatomical deviations (e.g. enlarged ventricles) that prevent appropriate image processing were additional exclusion criteria. |
| Münster Neuroimaging Cohort | SCID interview | MDD subjects: presence of bipolar disorder, schizoaffective disorders and schizophrenia; substancerelated disorders or current benzodiazepine treatment (wash out of at least three half-lives before study participation), and former electroconvulsive therapy, any neurological abnormalities, MRI contra-indications. |
| Novosibirsk | MINI, SCID, ICD-10 interviews | MDD subjects: Presence of axis-I disorders other than MDD, panic disorder, social anxiety disorder, or generalized anxiety disorder and any use of psychotropic medication other than stable use of SSRIs or infrequent benzodiazepine use; age 18 or below; alcohol or substance abuse/dependence within 6 months of study participation; current major medical problems; MRI contra-indications. |
| Oxford | SCID interview | MDD: psychosis or substance dependence (DSM-IV), clinically significant risk of suicidal behaviour, having contraindications to escitalopram treatment or being treated with psychotropic medication less than three weeks before the study (five weeks in the case of fluoxetine); major somatic or neurological disorders, pregnancy or breast-feeding, contra-indications to MR imaging or concurrent medication which could alter emotional processing. |
| Stanford | SCID interview | MDD subjects: presence of axis-I disorders other than MDD, anxiety and eating disorders; alcohol / substance abuse or dependence within six months prior to MRI scanning, history of head trauma with loss of consciousness > 5 min, aneurysm, or any neurological or metabolic disorders that require ongoing medication or that may affect the central nervous system (including thyroid disease, diabetes, epilepsy or other seizures, or multiple sclerosis), MRI contraindications, or bad MRI data (e.g., extreme movement). |
| Sydney | SCID interview | MDD subjects: presence of axis-I disorders other than MDD, panic disorder, social anxiety disorder, or generalized anxiety disorder; medical instability (as determined by a psychiatrist), history of neurological disease (e.g. tumour, head trauma, epilepsy), medical illness known to impact cognitive and brain function (e.g. cancer), intellectual and/or developmental disability and insufficient English for neuropsychological assessment. All subjects were asked to abstain from drug or alcohol use for 48 hours prior to testing and informed about a drug screen protocol. |
| Abbreviations: MDD: Major Depressive Disorder; CIDI: the Composite International Diagnostic Interview; SCID: Structured Clinical Interview for DSM disorders; SCAN: Schedules for Clinical Assessment in Neuropsychiatry; MINI: M.I.N.I. International Neuropsychiatric Interview; DSM: Diagnostic and Statistical Manual of Mental Disorders; MRI: Magnetic Resonance Imaging | | |

| Supplementary Table S2. Demographics and clinical characteristics of Bipolar Disorder patients | | | | | | | | | | |
| --- | --- | --- | --- | --- | --- | --- | --- | --- | --- | --- |
| Sample | N | Age | | % Male | HDRS-17 | | HDRS-14 | | HDRS Insomnia | |
|  |  | Mean | SD |  | Mean | SD | Mean | SD | Mean | SD |
| Houston I | 101 | 30.5 | 12.6 | 30.7 | 11.5 | 7.4 | 9.4 | 6.5 | 2.1 | 1.8 |
| Houston II | 14 | 35 | 10.2 | 35.7 | 10.9 | 8 | 8.9 | 6.9 | 2.1 | 2 |
| Münster Neuroimaging Cohort | 53 | 38 | 11.7 | 49.1 | 16.1 | 8.2 | 13.7 | 7.2 | 2.5 | 2 |
| FOR2017 - Münster | 25 | 41.7 | 13.2 | 72 | 7.7 | 5.1 | 6.4 | 4.9 | 1.2 | 1.7 |
| FOR2017 - Marburg | 48 | 43.7 | 10.8 | 35.4 | 6 | 6 | 5.2 | 5.4 | 0.9 | 1.3 |
| Edinburgh | 24 | 46.2 | 4.9 | 41.7 | 6 | 4.1 | 5.3 | 4.1 | 0.8 | 1 |
| Total | 265 | 37.1 | 12.9 | 40.4 | 10.6 | 7.8 | 8.8 | 6.8 | 1.8 | 1.8 |

| Supplementary Table S3. Demographics and characteristics of healthy controls | | | | | | |
| --- | --- | --- | --- | --- | --- | --- |
|  | N | Age | | % Male | Insomnia^a^ | |
|  |  | Mean | SD |  | Mean | SD |
| ENIGMA healthy controls | 1277 | 35.2 | 13.8 | 41.9% | 0.4 | 0.8 |
| HCP healthy controls | 831 | 28.8 | 3.7 | 48.4% | 2.2 | 1.6 |
| Note: ^a^ For the ENIGMA heathy controls insomnia severity is derived by summing up the scores of item 4, 5 and 6 of the Hamilton Depression Rating Scale, for the Human Connectome Project (HCP) healthy controls insomnia severity is the sum of the scores of item 5a and 5b of the Pittsburgh Sleep Quality Index (PSQI). | | | | | | |

| Supplementary Table S4. Image acquisition parameters by site | | | | | |
| --- | --- | --- | --- | --- | --- |
| Study | Scanner vendor and type | Acquisition parameters | FreeSurfer version | Slice orientation | Operating system |
| Barcelona | 3T Philips Achieva | 3D MPRAGE images (Whole-brain T1-weighted); TR=6.7ms, TE=3.2ms; 170 slices, voxel size 0.89X0.89X1.2 mm. Image dimensions 288X288X170; field of view: 256X256X204; slice thickness: 1.2 mm; with a sagittal slice orientation, T1 contrast enhancement, flip angle: 8º, grey matter as a reference tissue, ACQ matrix MXP = 256X240 and turbo-field echo shots (TFE) = 218. | 6.0 | Sagittal | scientific Linux 5 |
| Edinburgh | 1.5T GE Signa | T1-weighted sequence. TR=500 msec; TE=4 msec; flip angle 8°; matrix 192 x 192; 180 slices; voxel size 1.25 mm x 1.25 mm x 1.20 mm; FOV=24, phase FOV 1 | 5.3 | Coronal | linux 6, x86_64, kernel 2.6.32 |
| Calgary | 1.5T Siemens Magnetom Vision. 3T GE Discovery MR750 | 1.5T: A sagittal scout series was acquired to test image quality. 3D fast low angle shot (FLASH) sequence was used to acquire data from 124 1.5 mm-thick contiguous coronal slices through the entire brain (echo time = 5ms, repetition time = 25ms, acquisition matrix = 256 x 256 pixels, field of view = 24 cm and flip angle = 40°). 3T: Anatomical imaging acquisition parameters: axial acquisition, repetition time (TR), 2200 milliseconds (ms); echo time (TE), 3.04 ms; TI, 766, 780; flip angle, 13 degrees; 208 partitions; 256 × 256 matrix; and field of view, 256. | 5.3 | Coronal (1.5T), Axial (3T) | MacOs Sierra |
| CLING | 3T Siemens Tim Trio | T1-weighted 3D MPRAGE; TR/TE/TI/FA=2250 ms/3.26 ms/900 ms/9°; image matrix = 256 x 256; 192 sagittal slices; voxel size= 1 mm3 | 5.3 | sagittal | Linux |
| Dublin | 3T Phillips Achieva; 1.5T Siemens Vision | 3T: A sagittal T1 3D TFE was used to scan all participants. TR=8.5 msec; TE=3.9 msec; FOV = 256 mm, AP: 256 mm, RL: 160 mm; matrix: 256×256. 1.5T: 3D-MPRAGE T1-weighted sequence. TR=11.6 msec; TE=4.9 msec; FOV=230 mm; matrix 512 x 512, slice thickness: 1.5 mm. | 5.3 | Sagittal (3T), Coronal (1.5T) | Mac OS |
| FOR2107 Marburg | 3T Siemens Magnetom TiroTim syngo; | - Sequence: 3D T1-weighted magnetization prepared rapid acquisition gradient echo (MPRAGE) - Sagittal Acquisition Direction, # of Slices 176, 0.5mm Slice Gap, 1.0x1.0x1.0 Voxel Size (mm3), TI 900 ms, TE 2.26 ms, TR 1900 ms, Flip Angle 9. | 5.3 | Sagittal | Red Hat Enterprise Linux Server release 5.11 (Tikanga) |
| FOR2107 Münster | 3T Siemens PRISMA | - Sequence: 3D T1-weighted magnetization prepared rapid acquisition gradient echo (MPRAGE). - Sagittal Acquisition Direction, # of Slices 192, 0mm Slice Gap, 1.0x1.0x1.0 Voxel Size (mm3), TI 900 ms, TE 2.28 ms, TR 1900 ms, Flip Angle 8 | 5.3 | Sagittal | Red Hat Enterprise Linux Server release 5.11 (Tikanga) |
| Houston | 1.5 T Philips Medical Systems Gyroscan Intera | T -1 weighted fast field echo sequence (3D T1 -FFE) with repetition time (TR) = 25 ms, echo time (TE) = 5 ms, field of view (FOV) = 240 mm × 220 mm, gap = 0, and matrix size = 256 × 256. | 5.3 | Sagittal/ Transverse | Fedora 19 |
| Magdeburg | 3 Tesla Siemens MAGNETOM Trio scanner (Siemens, Erlangen, Germany) | High resolution T1 -weighted structural MRI scans of the brain were acquired for structural reference using a 3D -MPRAGE sequence (TE = 4.77 ms, TR = 2500 ms, T1 = 1100 ms, flip angle = 7°, bandwidth = 140 Hz/pixel, acquisition matrix = 256 × 256 × 192, isometric voxel size = 1.0 mm3). | 5.3 | Sagittal | Oracle Linux Server_x86_64 |
| MPIP | 1.5T GE and Siemens (the latter: only few cases) | #1: T1-weighted SPGR sagittal 3D volume. TR=1030 msec; TE=3.4 msec; 124 slices; matrix=256x256; FOV=23.0x23.0 cm2; voxel size=0.8975 mm x0.8975 mm x 1.2- 1.4 mm; flip angle=90°; birdcage resonator. #2: same scanner as #1, platform update Signa Excite, sagittal T1-weighted (spin echo sequence, TR=9.7 msec, TE=2.1 msec; FOV=25.0x25.0 cm2, voxel size=0.875 mm x0.875 mm x1.2 mm, 124- 132 slices, flip angle=90°. #3: Siemens 1.5 Tesla, Vario, 3D MPRAGE, TR=11.6 msec; TE=4.9 msec; FOV 23x23 cm2; matrix 512x512; 126 axial slices; voxel site 0.45 mm x 0.45 mm x 1.5 mm. (only N=2 subjects) | 5.3 | Sagittal (1.5 GE), axial (1.5 Siemens) | Linux 2.6.37.1-1.2- desktop |
| Münster Neuroimaging Cohort | 3T Philips Gyroscan Intera | 3D fast gradient echo sequence (turbo field echo), repetition time = 7.4 milliseconds, echo time = 3.4 milliseconds, flip angle = 9°, two signal averages, inversion prepulse every 814.5 milliseconds, acquired over a field of view of 256 (feet -head [FH]) × 204 (anterior -posterior [AP]) × 160 (right -left [RL]) mm, phase encoding in AP and RL direction, reconstructed to cubic voxels of .5 mm × .5 mm × .5 mm | 5.3 | Sagittal | Red Hat Enterprise Linux Server release 5.11 (Tikanga) |
| Novosibirsk | 3T GE Discovery™ MR750w | Whole-brain T1-weighted images - 3D fast spin gradient echo sequence (FSPGR BRAVO), repetition time = 9.5 ms, echo time = 3.7 ms, flip angle = 3°, acquired over a field of view of 256 (feet-head [FH]) × 256 (anterior-posterior [AP]) × 188 (rightleft [RL]) mm, reconstructed to cubic voxels of 1 mm × 1 mm × 1 mm | 5.3 | Sagittal | OS X 10.10 |
| Oxford | 3T Siemens Tim Trio | Voxel resolution 0.78 x 0.8 x 0.78 mm on a 208 x 256 x 200 grid, TE/TI/TR= 4.8/1100/2040 ms | 5.3 |  |  |
| Stanford | 1.5T GE Signa Excite | Whole-brain T1-weighted images were collected using a spoiled gradient echo (SPGR) pulse sequence (116 sagittal slices; through-plane resolution = 1.5 mm; in-plane resolution = 0.86 x 0.86 mm; flip angle = 15 degrees; repetition time [TR] = 8.3-10.1 ms; echo time [TE] = 1.7-3.0; inversion time [TI] = 300 ms; matrix = 256 x 192). | 5.3 | Sagittal | Linux-centos6_x86_64 |
| Sydney | 3T GE MR750 | 3D T1-weighted sequence. TR=7.2 msec; TE=2.78 msec; matrix =256; FOV=240; No. slices=196; thick=0.9mm; inplane resolution=0.9375 | 5.1 | Coronal | Linux_Ubuntu12.04_6 4 |
| HCP | 3T Siemens Skyra | voxel size = .7mm^3^ isotropic, Field of View = 224 mm, matrix = 320, 256 slices, repetition time (TR) = 2400ms, Echo Time (TE) = 2.14ms | 5.2 | Sagittal |  |
| Abbreviations: 3D: three-dimensional; TR: repetition time; TE: echo time; FOV: field of view | | | |  |  |

| Supplementary Table S5. Comparison of explained variance and Cohen’s f^2^ for the association with cortical surface area for insomnia severity, and depression severity | | | | |
| --- | --- | --- | --- | --- |
|  | Insomnia severity | | Depression severity (HDRS-17) | |
|  | ∆R^2^ (in %) | f^2^ | ∆R^2^ (in %) | f^2^ |
| Left inferior frontal gyrus pars triangularis | 1.8 | 0.023 | 0.3 | 0.006 |
| Right superior parietal cortex | 1.6 | 0.021 | -0.3 | -0.005 |
| Left frontal pole | 0.6 | 0.008 | -0.1 | -0.001 |
| Right medial orbitofrontal cortex | 1.3 | 0.016 | -0.2 | -0.003 |
| Right insula | 1.5 | 0.019 | 0.1 | 0.001 |
| Right supramarginal gyrus | 1.3 | 0.017 | 0.0 | 0.000 |
| Total cortical surface area | 0.9 | 0.012 | -0.3 | -0.005 |
| ∆R^2^ = The change in explained variance by adding the surface area of the brain region to a regression model containing only the covariates age, sex, and site as regressors (and HDRS-14 for the models with insomnia severity outcome measure). Negative values indicate that the model that includes the brain region explains less variance than the model with covariates only, i.e. the brain region only introduces additional noise. f^2^ = Cohen’s f^2^ statistic. Values of f^2^ = 0.02, 0.15 and 0.35, respectively, indicate a small, medium, or large effect. None of the surface areas significantly contribute to explaining overall depression severity as measured with the complete Hamilton Depression Rating Scale (HDRS-17). | | | | |

| Supplementary Table S6. Mixed effect regression analyses estimates of the association of insomnia severity with cortical thickness (HDRS points/mm) in MDD patients, adjusted for age, sex, insomnia-independent depression severity^a^ and scanning site | | | | | | | |
| --- | --- | --- | --- | --- | --- | --- | --- |
|  | *B* | *s.e.* | *95% CI* | *t-value* | *p-value* | *FDR p-value* | *N* |
| Right inferior frontal gyrus pars orbitalis | 0.61 | 0.24 | 0.14 to 1.08 | 2.54 | 0.011 | 0.574 | 1040 |
| Left temporal pole | 0.33 | 0.14 | 0.06 to 0.60 | 2.39 | 0.017 | 0.574 | 1024 |
| Left inferior frontal gyrus pars orbitalis | 0.48 | 0.24 | 0.00 to 0.96 | 1.95 | 0.051 | 0.574 | 1041 |
| Right parahippocampal gyrus | 0.33 | 0.18 | -0.04 to 0.69 | 1.76 | 0.078 | 0.574 | 1037 |
| Right lateral orbitofrontal cortex | 0.51 | 0.30 | -0.08 to 1.10 | 1.71 | 0.088 | 0.574 | 1047 |
| Right entorhinal cortex | 0.25 | 0.15 | -0.04 to 0.54 | 1.68 | 0.093 | 0.574 | 810 |
| Right superior parietal cortex | 0.69 | 0.42 | -0.13 to 1.51 | 1.66 | 0.097 | 0.574 | 1032 |
| Right cuneus | 0.57 | 0.37 | -0.14 to 1.29 | 1.57 | 0.117 | 0.574 | 1017 |
| Left transverse temporal gyrus | 0.39 | 0.25 | -0.10 to 0.87 | 1.57 | 0.118 | 0.574 | 1050 |
| Right postcentral gyrus | 0.69 | 0.44 | -0.18 to 1.55 | 1.56 | 0.119 | 0.574 | 1035 |
| Left banks superior temporal sulcus | 0.51 | 0.33 | -0.14 to 1.16 | 1.55 | 0.121 | 0.574 | 963 |
| Right inferior frontal gyrus pars opercularis | 0.54 | 0.35 | -0.15 to 1.23 | 1.53 | 0.127 | 0.574 | 1018 |
| Right inferior frontal gyrus pars triangularis | 0.56 | 0.36 | -0.16 to 1.27 | 1.53 | 0.127 | 0.574 | 1017 |
| Right precentral gyrus | 0.59 | 0.39 | -0.18 to 1.36 | 1.50 | 0.134 | 0.574 | 1043 |
| Right isthmus cingulate cortex | -0.40 | 0.27 | -0.93 to 0.13 | -1.50 | 0.135 | 0.574 | 1047 |
| Left posterior cingulate cortex | -0.50 | 0.33 | -1.15 to 0.16 | -1.49 | 0.138 | 0.574 | 1046 |
| Left fusiform gyrus | 0.47 | 0.32 | -0.16 to 1.10 | 1.46 | 0.144 | 0.574 | 1043 |
| Left parahippocampal gyrus | 0.24 | 0.16 | -0.09 to 0.56 | 1.43 | 0.153 | 0.574 | 1036 |
| Right rostral middle frontal gyrus | 0.53 | 0.38 | -0.21 to 1.28 | 1.40 | 0.160 | 0.574 | 1034 |
| Right supramarginal gyrus | 0.54 | 0.42 | -0.29 to 1.38 | 1.29 | 0.199 | 0.579 | 976 |
| Right posterior cingulate cortex | -0.43 | 0.34 | -1.09 to 0.24 | -1.26 | 0.208 | 0.579 | 1048 |
| Left lateral occipital cortex | 0.51 | 0.42 | -0.30 to 1.33 | 1.24 | 0.217 | 0.579 | 1037 |
| Left superior temporal gyrus | 0.42 | 0.35 | -0.26 to 1.11 | 1.20 | 0.229 | 0.579 | 917 |
| Left cuneus | 0.46 | 0.39 | -0.29 to 1.22 | 1.20 | 0.231 | 0.579 | 1010 |
| Left lateral orbitofrontal cortex | 0.37 | 0.31 | -0.24 to 0.98 | 1.19 | 0.235 | 0.579 | 1045 |
| Left precentral gyrus | 0.48 | 0.40 | -0.32 to 1.27 | 1.18 | 0.238 | 0.579 | 1033 |
| Left middle temporal gyrus | 0.38 | 0.33 | -0.26 to 1.02 | 1.16 | 0.246 | 0.579 | 978 |
| Left caudal middle frontal gyrus | 0.40 | 0.35 | -0.29 to 1.09 | 1.13 | 0.258 | 0.579 | 1036 |
| Left superior parietal cortex | 0.49 | 0.43 | -0.36 to 1.34 | 1.13 | 0.259 | 0.579 | 1027 |
| Left rostral middle frontal gyrus | 0.41 | 0.37 | -0.31 to 1.13 | 1.13 | 0.259 | 0.579 | 1026 |
| Right transverse temporal gyrus | 0.27 | 0.24 | -0.20 to 0.74 | 1.12 | 0.264 | 0.579 | 1051 |
| Left inferior frontal gyrus pars opercularis | 0.39 | 0.37 | -0.34 to 1.11 | 1.04 | 0.300 | 0.638 | 1034 |
| Left inferior frontal gyrus pars triangularis | 0.34 | 0.35 | -0.35 to 1.03 | 0.96 | 0.335 | 0.668 | 1032 |
| Right temporal pole | 0.12 | 0.13 | -0.13 to 0.38 | 0.95 | 0.340 | 0.668 | 1028 |
| Left precuneus | 0.38 | 0.41 | -0.43 to 1.20 | 0.93 | 0.353 | 0.668 | 1042 |
| Right precuneus | 0.38 | 0.40 | -0.42 to 1.17 | 0.93 | 0.354 | 0.668 | 1046 |
| Left postcentral gyrus | 0.38 | 0.44 | -0.49 to 1.26 | 0.86 | 0.388 | 0.698 | 1028 |
| Left insula | 0.30 | 0.34 | -0.38 to 0.97 | 0.86 | 0.390 | 0.698 | 1029 |
| Right lateral occipital cortex | 0.31 | 0.39 | -0.45 to 1.07 | 0.79 | 0.427 | 0.698 | 1039 |
| Left pericalcarine cortex | 0.28 | 0.36 | -0.42 to 0.98 | 0.79 | 0.432 | 0.698 | 1013 |
| Right banks superior temporal sulcus | -0.23 | 0.30 | -0.81 to 0.35 | -0.77 | 0.440 | 0.698 | 987 |
| Left caudal anterior cingulate cortex | 0.16 | 0.21 | -0.25 to 0.58 | 0.77 | 0.442 | 0.698 | 1045 |
| Left paracentral lobule | 0.28 | 0.37 | -0.44 to 1.01 | 0.77 | 0.444 | 0.698 | 1047 |
| Left inferior temporal gyrus | 0.22 | 0.30 | -0.37 to 0.82 | 0.74 | 0.461 | 0.698 | 1009 |
| Right rostral anterior cingulate cortex | 0.16 | 0.23 | -0.28 to 0.61 | 0.73 | 0.465 | 0.698 | 1035 |
| Left superior frontal gyrus | 0.27 | 0.37 | -0.46 to 1.00 | 0.72 | 0.472 | 0.698 | 1034 |
| Right superior frontal gyrus | 0.26 | 0.37 | -0.47 to 0.99 | 0.69 | 0.488 | 0.707 | 1044 |
| Right paracentral lobule | 0.23 | 0.36 | -0.48 to 0.94 | 0.64 | 0.521 | 0.739 | 1046 |
| Right caudal middle frontal gyrus | 0.23 | 0.37 | -0.50 to 0.95 | 0.62 | 0.535 | 0.743 | 1039 |
| Right superior temporal gyrus | 0.20 | 0.36 | -0.50 to 0.91 | 0.57 | 0.569 | 0.765 | 917 |
| Right lingual gyrus | -0.23 | 0.41 | -1.03 to 0.58 | -0.55 | 0.582 | 0.765 | 1035 |
| Left rostral anterior cingulate cortex | 0.12 | 0.23 | -0.32 to 0.56 | 0.55 | 0.585 | 0.765 | 1032 |
| Right caudal anterior cingulate cortex | 0.12 | 0.22 | -0.32 to 0.55 | 0.52 | 0.600 | 0.770 | 1045 |
| Left inferior parietal cortex | 0.21 | 0.42 | -0.61 to 1.02 | 0.50 | 0.618 | 0.779 | 1011 |
| Right inferior parietal cortex | 0.19 | 0.41 | -0.61 to 1.00 | 0.48 | 0.634 | 0.784 | 1018 |
| Right medial orbitofrontal cortex | 0.12 | 0.30 | -0.46 to 0.71 | 0.41 | 0.680 | 0.826 | 1030 |
| Left medial orbitofrontal cortex | 0.11 | 0.31 | -0.49 to 0.72 | 0.37 | 0.708 | 0.845 | 1023 |
| Left lingual gyrus | 0.14 | 0.42 | -0.67 to 0.96 | 0.35 | 0.729 | 0.854 | 1045 |
| Right fusiform gyrus | 0.07 | 0.30 | -0.52 to 0.67 | 0.24 | 0.810 | 0.933 | 1041 |
| Left entorhinal cortex | -0.03 | 0.16 | -0.34 to 0.28 | -0.19 | 0.847 | 0.953 | 851 |
| Left isthmus cingulate cortex | -0.04 | 0.26 | -0.55 to 0.47 | -0.17 | 0.866 | 0.953 | 1043 |
| Right insula | 0.05 | 0.32 | -0.57 to 0.68 | 0.16 | 0.869 | 0.953 | 1015 |
| Left frontal pole | 0.02 | 0.18 | -0.32 to 0.37 | 0.13 | 0.894 | 0.956 | 1051 |
| Right frontal pole | -0.02 | 0.19 | -0.39 to 0.34 | -0.12 | 0.904 | 0.956 | 1051 |
| Right pericalcarine cortex | 0.04 | 0.36 | -0.66 to 0.74 | 0.11 | 0.914 | 0.956 | 1012 |
| Right middle temporal gyrus | 0.02 | 0.31 | -0.59 to 0.64 | 0.08 | 0.938 | 0.961 | 1011 |
| Left supramarginal gyrus | 0.02 | 0.42 | -0.80 to 0.85 | 0.05 | 0.959 | 0.961 | 959 |
| Right inferior temporal gyrus | 0.01 | 0.29 | -0.55 to 0.58 | 0.05 | 0.961 | 0.961 | 1027 |
| Abbreviations: MDD, Major Depressive Disorders; HDRS, Hamilton Depression Rating Scale; CI, confidence interval; FDR, false-discovery rates; ^a^ insomnia-independent depression severity is calculated by subtracting the insomnia scores from the total HDRS score; | | | | | | | |

| Supplementary Table S7. Mixed effect regression analyses estimates of the association of insomnia severity with subcortical volumes (HDRS points/cm^3^) in MDD patients, adjusted for age, sex, insomnia-independent depression severity^a^ and scanning site | | | | | | | |
| --- | --- | --- | --- | --- | --- | --- | --- |
|  | *B* | *s.e.* | *95% CI* | *t-value* | *p-value* | *FDR p-value* | *N* |
| Left lateral ventricle | -0.01 | 0.01 | -0.04 to 0.01 | -1.08 | 0.281 | 0.886 | 1046 |
| Left pallidum | 0.20 | 0.24 | -0.26 to 0.67 | 0.86 | 0.389 | 0.886 | 957 |
| Left caudate | 0.10 | 0.13 | -0.15 to 0.34 | 0.78 | 0.436 | 0.886 | 1025 |
| Left accumbens | 0.39 | 0.50 | -0.59 to 1.37 | 0.78 | 0.437 | 0.886 | 1029 |
| Left amygdala | 0.18 | 0.27 | -0.34 to 0.71 | 0.68 | 0.496 | 0.886 | 1030 |
| Right lateral ventricle | -0.01 | 0.01 | -0.04 to 0.02 | -0.67 | 0.502 | 0.886 | 1045 |
| Right putamen | -0.06 | 0.09 | -0.24 to 0.13 | -0.62 | 0.537 | 0.886 | 1007 |
| Right accumbens | -0.26 | 0.58 | -1.39 to 0.88 | -0.45 | 0.654 | 0.886 | 1030 |
| Right thalamus | -0.03 | 0.08 | -0.19 to 0.13 | -0.38 | 0.703 | 0.886 | 1034 |
| Left thalamus | -0.02 | 0.06 | -0.15 to 0.10 | -0.37 | 0.708 | 0.886 | 1028 |
| Right pallidum | 0.09 | 0.28 | -0.46 to 0.63 | 0.32 | 0.751 | 0.886 | 1017 |
| Right caudate | 0.04 | 0.12 | -0.20 to 0.28 | 0.31 | 0.755 | 0.886 | 1025 |
| Right hippocampus | 0.04 | 0.13 | -0.21 to 0.29 | 0.31 | 0.759 | 0.886 | 1034 |
| Left putamen | -0.02 | 0.08 | -0.19 to 0.14 | -0.29 | 0.775 | 0.886 | 989 |
| Left hippocampus | 0.02 | 0.11 | -0.20 to 0.23 | 0.17 | 0.864 | 0.898 | 1034 |
| Right hippocampus | -0.03 | 0.24 | -0.51 to 0.45 | -0.13 | 0.898 | 0.898 | 1035 |
| Abbreviations: MDD, Major Depressive Disorders; HDRS, Hamilton Depression Rating Scale; CI, confidence interval; FDR, false-discovery rates; ^a^ insomnia-independent depression severity is calculated by subtracting the insomnia scores from the total HDRS score; | | | | | | | |

Supplementary Figure S1. T-scores for all regions (both significant and non-significant regions) that show the association between surface area and insomnia severity scores in Major Depressive Disorder patients. Models are adjusted for age, sex, insomnia-independent depression severity (HDRS-14) and site. RH = right hemisphere, LF= left hemisphere.

**References**

1. Van Essen D. C. *et al.* The WU-Minn Human Connectome Project: an overview. *Neuroimage* 2013; **80:** 62-79.

2. Buysse D. J., Reynolds III C. F., Monk T. H., Berman S. R., Kupfer D. J. The Pittsburgh Sleep Quality Index: a new instrument for psychiatric practice and research. *Psychiatry Res* 1989; **28**(2)**:** 193-213.

3. Bucholz K. K. *et al.* A new, semi-structured psychiatric interview for use in genetic linkage studies: a report on the reliability of the SSAGA. *Journal of Studies on Alcohol* 1994; **55**(2)**:** 149-158.

4. Glasser M. F. *et al.* The minimal preprocessing pipelines for the Human Connectome Project. *Neuroimage* 2013; **80:** 105-124.

5. Manber R. *et al.* Assessing insomnia severity in depression: comparison of depression rating scales and sleep diaries. *J Psychiatr Res* 2005; **39**(5)**:** 481-488.

6. Watanabe N. *et al.* Brief behavioral therapy for refractory insomnia in residual depression: An assessor-blind, randomized controlled trial. *The Journal of clinical psychiatry* 2011; **72**(12)**:** 1651-1658.

7. Enneking V. *et al.* Social anhedonia in major depressive disorder: a symptom-specific neuroimaging approach. *Neuropsychopharmacol* 2019; **44**(5)**:** 883-889.

8. Achenbach T. M. *The Achenbach system of empirically based assessment (ASEBA): Development, findings, theory, and applications*. University of Vermont, Research Center for Children, Youth, & Families2009.
